# Farming Practices Influence Antibiotic Resistance and Biogenic Amine Capacity of Staphylococci from Bulk Tank Ewe’s Milk

**DOI:** 10.3390/ani10091622

**Published:** 2020-09-10

**Authors:** Justa María Poveda, Lorena Jiménez, José Manuel Perea, Ramón Arias, María Llanos Palop

**Affiliations:** 1 Department of Analytical Chemistry and Food Technology, Regional Institute of Applied Scientific Research (IRICA)/Faculty of Chemical Sciences and Technologies, University of Castilla-La Mancha, Camilo José Cela, 1B, 13071 Ciudad Real, Spain; Justamaria.poveda@uclm.es; 2 Regional Center of Animal Selection and Reproduction (CERSYRA), Agri-food and Forestry Regional Research and Development Center (IRIAF), JCCM, 13300 Valdepeñas, Spain; ljimenez@quesomanchego.es; 3 Department of Animal Production, Faculty of Veterinary, University of Cordoba, Campus Rabanales, 14071 Córdoba, Spain; pa2pemuj@uco.es; 4 Department of Analytical Chemistry and Food Technology, Faculty of Environmental Sciences and Biochemistry, University of Castilla-La Mancha, Avda. Carlos III, s/n, 45071 Toledo, Spain; MariaLlanos.Palop@uclm.es

**Keywords:** staphylococci, ewe’s milk, antibiotic resistance, biogenic amine, farming practices

## Abstract

**Simple Summary:**

Staphylococci are one of the main microorganisms responsible for intramammary infections in sheep—a syndrome causing important economic losses to farmers. Additionally, eventual staphylococcal contamination of milk could cause significant health problems in humans, especially by the consumption of dairy products made with raw milk containing toxic compounds, such as biogenic amines or antibiotic resistant bacteria. This study aims to check the presence and safety of staphylococci in bulk tank ewe’s milk from different farms, and to determine the relationship between the presence of these staphylococci with farming practices. We found that among the indigenous staphylococcal population in bulk tank ewe’s milk, there were multidrug-resistant and aminobiogenic isolates. Additionally, it was observed that some farming practices, especially those related to the hygienic-sanitary conditions, affect the risk of finding antibiotic-resistant and biogenic amine-producing *Staphylococcus* isolates in milk. Therefore, farmers should make stronger efforts not only to prevent animals from suffering an intramammary infection but also to avoid compromising consumers’ health and to stop the spread of antibiotic-resistant bacteria.

**Abstract:**

Staphylococci are one of the main microorganisms responsible for intramammary infections in sheep, causing important economic losses for farmers and eventually health problems in humans, especially by the consumption of dairy products made with raw milk containing toxic compounds, such as biogenic amines or antibiotic resistant bacteria. This study aimed to check the presence and safety of staphylococci in bulk tank ewe’s milk from different farms, and to determine the relationship between the presence of these staphylococci and farming practices, by applying nonlinear canonical correlation models (OVERALS). Two-hundred and fifty-nine staphylococci from milk samples from eighteen farms were genotyped and representative isolates of the major clusters were identified as belonging to *Staphylococcus (S.) aureus*, *S. epidermidis*, *S. arlettae*, *S. lentus*, *S. simulans*, and *S. chromogenes* species. Identified isolates were assayed in terms of their safety, by evaluating resistance to antimicrobial drugs and the aminobiogenic capacity, using both phenotypic and genetic assays. Antibiotic resistance phenotypic assay revealed that 82.9% were resistant to some antibiotics, although in the genotypic assay only the genes *tet*M, *erm*B, *erm*C, and *grl*A were detected. Fifty-three percent were high biogenic amine (BA) producers, being putrescine the most produced amine. A lowered risk of finding antibiotic-resistant and BA-producing staphylococci is related to some farming methods such as enrolling in a breeding program, use of good farming practices, postdipping teat disinfection, hygienic livestock housing, or periodic check of the milking machine.

## 1. Introduction

Staphylococci are one of the main microorganisms responsible for intramammary infections in ewes—a syndrome causing significant economic losses to farmers by decreased milk yields, death and early culling of animals, or the necessary administration of antibiotics [1]. Different species have been reported to be present in these infections, and while *Staphylococcus (S.) aureus*, a coagulase-positive staphylococci (CPS) species, is frequently isolated from clinical mastitis, coagulase-negative staphylococci (CNS) are more frequent in subclinical mastitis, the main type of intramammary infections in ewes [2]. In addition to the above-mentioned problems, another important aspect that should be considered is that eventual staphylococcal contamination of milk could cause significant health problems in humans, especially by the consumption of dairy products made with raw milk [3], including those related to antibiotic resistance transmission between bacteria or the presence of biogenic amines (BA) in milk [4].

BAs are low-molecular weight nitrogenous compounds that are formed in foodstuffs by microbial decarboxylation of the precursor amino acids. Its presence in high amounts in food and beverages has traditionally been used as an indicator of their hygienic quality and degree of microbial alteration [5,6]. The importance of the presence of BA in food lies in their potential toxicity to humans, causing problems for susceptible consumers such as headaches, vertigo, nausea and vomiting, and increase in arterial blood pressure [7]. Since the ability of microorganisms to decarboxylate amino acids is highly variable, being in most cases a strain-dependent property, the detection of bacteria possessing this activity in raw milk is relevant because it could have an important impact in the quality of artisanal cheese. In fact, some studies on raw milk Manchego cheese corroborate the presence of BAs in high quantities [8,9]. On the other hand, it is well known that mastitis can affect quality and enhance proteolysis in milk, and some authors have found a relationship between the somatic cell counts in milk and the content of biogenic amines in cheeses made with that milk [10].

Antimicrobial resistance to antibiotics is a growing health problem worldwide, and actions to restrict the use in animals of antimicrobials that are of critical importance for preventing or treating life-threatening infections in humans have been implemented. In this respect, Regulation EU no 2019/16 [11] includes the strengthening of the prudent use of antimicrobials in veterinary science, avoiding their routine prophylactic and metaphylactic use. In addition, in order to use antimicrobials, the corresponding veterinary prescription, after the realization of antimicrobial susceptibility tests, is mandatary, being necessary to communicate it to the competent authority.

European Union legislation provides microbiological criteria for foods of animal origin (Commission Regulation EC no 2073/2005 [12]), establishing limits for coagulase-positive staphylococci in cheese and other dairy products (‘process hygiene criterion’), and recommending to take measures to improve production hygiene and the selection of raw milk when the limits are exceeded. However, limits for these bacteria in milk have not been set.

There are studies in sheep milk that link microbial contamination of milk with some farm management practices, such as hygienic conditions and milking system [13,14], while others focus on measures aimed at the prevention and control of mastitis caused by staphylococci, among other microorganisms [1]. However, there are no studies showing the relationship between farm management practices and staphylococcal contamination in milk, along with some of the collateral problems resulting from this contamination, such as the increase in antibiotic-resistant bacteria or BA production.

The aims of this study were (1) to check the microbiological quality of bulk tank ewe’s milk from different farms, in terms of the presence and safety of the staphylococcal population, by isolating, genotyping, and identifying staphylococci in samples, and by evaluating resistance to antimicrobial drugs and the aminobiogenic capacity of the isolates identified, using both phenotypic and genetic assays; (2) to determine the relationship between farming practices and resistance to antimicrobial drugs or BA production of isolated staphylococci.

## 2. Materials and Methods

### 2.1. Population and Study Design

Eighteen typical farms with dairy systems representative of the main milking and feeding practices in Castilla-La Mancha region were selected for sampling. Farmers were asked about their flock size, general management, milking practices, milk production, and sanitary conditions of the flocks, including their enrolment (or not) in a breeding program. In addition, the handling and characteristics of the milking machines, the hygienic conditions of milking parlor and livestock housing, and the implementation (or not) of a guide to good farming practices, postdipping teat disinfection, and dry-off treatments were referenced.

### 2.2. Sampling, Bacterial Isolation, and Genotyping and Identification of the Isolates

A total of 36 samples of bulk tank ewe’s milk (two from each selected farm) were taken from dairy flocks throughout one year. In each sampling, 100 mL milk was taken under aseptic conditions, which was maintained refrigerated while transported to the laboratory and immediately analyzed. Samples were serially diluted in sterile saline solution and spread in duplicate into Baird Parker Agar (BPA) (BioMérieux, Madrid, Spain) plates that were incubated at 37 °C for 24 h in aerobic conditions. Counts were expressed as CFU/mL of milk. From countable BPA plates, seven to eight isolated colonies were randomly picked and purified by successive streaking on the same medium. Pure cultures were stored at −80 °C in Brain Heart Infusion (BHI) broth (Oxoid Limited, Basingstoke, UK) containing 20% (*v*/*v*) glycerol (Panreac).

Genotyping was carried out by using Pulsed Field Gel electrophoresis (PFGE). Preparation of intact genomic DNA was performed by a modified version of the method described by Bannerman et al. [15]. Chromosomal DNA was digested with the endonuclease *Sma*I (New England Biolabs, Ipswich, MA, USA) at 37 °C for 16 h. Electrophoresis was carried out in a CHEF DR-III apparatus (Bio-Rad, Richmond, CA, USA) for 23 h at 14 °C at 6 V/cm with pulses from 5 to 35 s. A standard pattern (Lambda Ladder PFG Marker, New England Biolabs, Ipswich, MA, USA) was included in the gels for comparison of the digitally normalized PFGE pattern profiles. Computer-assisted analysis was performed with Phoretix 1D Pro software (Nonlinear Inc., Durham, NC, USA). Isolates were grouped using the Pearson-product moment correlation coefficient and cluster analysis by the unweighted pair group method with arithmetic average (UPGMA) [16].

A reproducibility study was carried out on four isolates and four iterations of the entire procedure in order to determine the minimum percentage similarity necessary for strain discrimination. Four separate cultures of each isolate were grown, and their DNA extracts were digested separately. The products obtained for two replicates of each isolate were run on one gel, and those for the other two replicates were run on another gel. Patterns were analyzed as described above, and the level of similarity observed between repeats established a discrimination threshold, below which patters were deemed different.

Representative isolates from clusters obtained in the numerical analysis of PFGE profiles were identified by Matrix Assisted Laser desorption/ionization-time of flight (MALDI-TOF) mass spectrometry in a Vitek-MS instrument (BioMérieux, Marcy-L’Etoile, France) following the protocol described by Jiménez et al. [17]. For each isolate, a mean spectrum was constructed with at least 50 *m*/*z* spectra profiles and used for the identification by comparison with the spectra contained in the Myla database (BioMérieux, Marcy-L’Etoile, France). Identification was defined as a 99% to 100% match to the species-specific *m*/*z* values in the database.

### 2.3. Antibiotic Resitance Assay

To analyze the antibiotic resistance of the *Staphylococcus* isolates, both a phenotypic and a genetic assay, for detection of selected resistance genes, were carried out.

For the phenotypic assay, the disk diffusion method on Mueller-Hinton agar plates, according to the guidelines of the Clinical and Laboratory Standards Institute (CLSI) [18], was used. Discs of the following antibiotics were used: penicillin (10 μg/disc), ampicillin (10 μg/disc), vancomycin (30 μg/disc), tetracycline (30 μg/disc), streptomycin (10 μg/disc), gentamicin (10 μg/disc), chloramphenicol (30 μg/disc), erythromycin (15 μg/disc), ciprofloxacin (5 μg/disc), clindamycin (2 μg/disc), and nitrofurantoin (300 μg/disc). All were purchased from Bio-Rad (Marnes-la-Coquette, Paris, France).

Isolates were grown on Tryptic Soy Agar (TSA) (Scharlab, Barcelona, Spain) plates at 37 ºC for 24 h and after incubation, a cell suspension in sterile saline solution with an optical density at 600 nm (OD600) equivalent to 0.5 of the McFarland scale was prepared. Then, the procedure reported by Ruiz et al. [19] was followed, and the isolates were classified as sensitive, intermediate resistant, or resistant according to the breakpoints reported by the CLSI [18]. Analyses were carried out in duplicate.

For the genetic assay, PCR amplifications of some structural genes associated with resistance to β-lactams (*bla*TEM, *oxa*, *mec*A), glycopeptides (*van*A), tetracyclines (*tet*M), quinolones (*gyr*A, *grl*A), aminoglycosides (*aac6′/aph2*″), and macrolides (*erm*A, *erm*B, *erm*C, *mef*A, *msr*A) were performed using the primers listed in Table 1. The thermal cycling program was that reported by Even et al. [20], using the annealing temperature in Table 1. PCR products were resolved by electrophoresis in 2% (*w*/*v*) agarose gel in 0.5x TBE (Tris-boric acid-EDTA) buffer. Primers TStaG422 (5′-GGCCGTGTTGAACGTGGTCAAATCA-3′) and TStag765 (5′-TIACCATTTCAGTACCTTCTGGTAA-3′) [21] targeting the *tuf* gene, which is present in all staphylococcal species, were added to the PCR reactions as a positive control.

### 2.4. Biogenic Amine Production

Before analysis, and to promote enzyme induction, isolates were cultivated six times in BHI broth added with 0.1% (*w*/*v*) of each precursor amino acid (L-histidine monohydrochloride, L-ornithine monohydrochloride, tyrosine disodium salt, and L-lysine monohydrochloride) purchased from Sigma (St. Louis, MO, USA) and 0.005% (*w*/*v*) of pyridoxal-5-phosphate, and incubated at 37 °C for 48 h. Cultures were centrifugated (15,000× *g*, 5 min, 4 °C), and cell-free supernatants were used for the quantitative assay of BA production by RP-HPLC. Noninoculated medium was used as a control. Isolates were assayed in triplicate.

The quantification of seven BAs (histamine, tyramine, putrescine, cadaverine, tryptamine, 2-phenylethylamine, and spermidine) was carried out by RP-HPLC using a diethyl ethoxymethylenemalonate (DEEMM) derivatization method [22].

In addition, the determination of amino acid decarboxylase genes was carried out by PCR reactions. Simultaneous detection of tyrosine decarboxylase (*tdc*), histidine decarboxylase (*hdc*), and ornithine decarboxylase (*odc*) genes was carried out using the conditions described by Coton et al. [23] and the primers listed in Table 1. The detection of the lysine decarboxylase (*ldc*) gene was performed using the conditions described by de las Rivas et al. [24], and the primers are listed in the same Table. The *tuf* gene was used as a positive control.

### 2.5. Statistical Analysis

One-way analysis of variance (ANOVA) was applied to the results, using the Student–Newman–Keuls (S–N–K) test for comparison of the means (*p* < 0.05). Hierarchical cluster analysis regarding the BA profiles was performed using Ward’s method and squared Euclidean distance.

For the study of the influence of the characteristics of livestock, three groups of variables were defined: farming characteristics, antibiotic resistance, and BA production. The farming practices evaluated were the following: flock size (Size: >1300 ewes or <1300 ewes), breeding program (Breeding P: enrolled or not enrolled), application of a guide to good farming practices (Guide FP: yes or not), hygiene of the livestock housing (Hyg LH: adequate or not), hygiene of the milking parlour (Hyg MP: adequate or not), milkline of the milking machine (Milkline: low-level or mid-level system), milking stalls (M Stalls: >48 or <48), ewes per milking stall (E/M Stall: >30 or <30), periodic check-up of the milking machine (Revision MM: yes or not), frequency of use of acid for cleaning the milking machine (Acid: daily or less frequently), mastitis vaccination (M vaccination: yes or not), postdipping teat disinfection (Postdipping TD: yes or not), and dry-off treatment (Dry-off T: yes or not).

The antibiotic resistance group of staphylococci was configured by the following variables: ampicillin (AMP: + or −), penicillin (PEN: + or −), vancomycin (VAN: + or −), tetracycline (TET: + or −), ciprofloxacin (CIP: + or −), streptomycin (SMN: + or −), gentamicin (GMN: + or −), clindamycin (CMN: + or −), erythromycin (ERY: + or −), nitrofurantoin (FTN: + or −), and chloramphenicol (CHL: + or −).

The group of amines of staphylococci was configured by the presence or absence of the following amines in tank milk: histamine (HIS: + or −), tyramine (TYR: + or −), putrescine (PUT: + or −), tryptamine (TRP: + or −), cadaverine (CAD: + or −), 2-phynylethylamine (2-PHA: + or −), and spermidine (SPD: + or −).

Nonlinear canonical correlation models (OVERALS) were applied to analyze the complex relationships between farming practices and antibiotic resistance and BA production by staphylococci. The purpose of OVERALS is to determine how similar the sets of categorical variables are to each other. OVERALS uses optimal scaling, which quantifies categorical variables and then treats them as numerical variables, including applying nonlinear transformations to find the best-fitting model [25]. The aim is to account for as much of the variance in the relationships among the sets as possible, in a low dimensional space, and to establish the similarities between the sets by simultaneously comparing linear combinations of the variables in each set to an unknown set. The variables in each set are linearly combined so that the linear combinations have a maximal correlation. Given these combinations, subsequent linear combinations are determined, which are uncorrelated with the previous combinations and that have the largest possible correlation. OVERALS searches for a subspace that several sets of variables, measured on the same objects, have in common. A perfect adaptation would comply with the number of chosen dimensions, where the maximum number of dimensions matches the sum of all linear combinations of variable characteristics from the sets. The loss function states the difference between the number of chosen dimensions to the best calculated adaptation [26]. Moreover, eigenvalues can be determined by analyzing data from the fit and loss function. These eigenvalues indicate to what extent every single dimension account for the loss function compared to the calculated correlation and can take on values between zero and one [27].

We conducted a manual model-building selection for the development of the OVERALS models [28]. As a first step, we compared all the possible models with just two variables per set using the fit value and the component loadings of variables. To the model with the highest fit value and at least a component loading greater than 0.4 in each variable, we added all the remaining variables one by one and compared them based on the fit value and the component loadings. This process was repeated until the model with the highest fit value was obtained. This was considered the most plausible and selected as the final model.

All statistical analyses were performed using the IBM SPSS statistics package version 24.0 (SPSS Inc., Chicago, IL, USA) [29].

## 3. Results

### 3.1. Isolation, Genotyping, and Identification of Isolates

Growth on BPA plates was observed from all the samples with counts ranging between 2.53 and 5.54 log CFU/mL (mean value of 4.38 ± 0.49 log CFU/mL). A total of 259 colonies were picked from countable plates and streaked on the same medium until purification.

PFGE analysis of pure isolates with the restriction enzyme SmaI, and cluster analysis by UPGMA of the patterns obtained, displayed 92 genotypes, defined at a minimum similarity value of 85%, the value obtained in the reproducibility study. Two-hundred and two isolates were grouped at 35 major clusters, which comprised two or more isolates from samples taken from the same or different dairy flocks, and the remaining isolates showed singular patterns (data not shown).

One representative isolate from each major cluster was identified by MALDI-TOF, revealing that 62.9% belonged to *S. aureus*, and the remaining were CNS of the following species: *S. epidermidis* (17.1%), *S. arlettae* (8.6%), *S. lentus*, *S. haemolyticus*, *S. simulans,* and *S. chromogenes*, which were represented at the same percentage (2.85% each). Isolates identified were used for the following assays.

### 3.2. Antibiotic Resistance Assays

Table 2 summarizes results from phenotypic and genetic antibiotic resistance assays. All the isolates were susceptible to gentamicin and vancomycin, while 94.3% were susceptible or intermediate susceptible to nitrofurantoin, clindamycin, and chloramphenicol.

It is remarkable that 82.9% of the isolates showed some resistance to nine of the 11 antibiotics assayed, with the antibiotics β-lactams (ampicillin and penicillin) and tetracycline showing the highest number of resistant isolates; 57.1% were resistant to ampicillin, 48.6% to penicillin, and 31.4% to tetracycline.

The incidence of resistance to antibiotics varied with the species and the isolate. The MAR (Multiple Antimicrobial Resistance) index [30] (Table 3)—defined as the ratio between the number of antimicrobials to which the isolate is resistant and the number of antimicrobials assayed—revealed that 54.3% of the isolates were resistant to two or more antibiotics, with the highest value being for the isolates Sa06 and Sa12, which were resistant to six of the 11 antibiotics assayed, followed by Se04 and Sc01, which were resistant to four antibiotics. Isolates Sa05 and Sa20 were the only ones susceptible to all the antibiotics assayed, while isolates Sa02, Sa04, Sa14, and Sa15 were susceptible or intermediate.

The comparison of results for the different species revealed that *S. aureus* isolates showed a higher variability in the antibiotic resistance profiles, ranging between one and six antibiotics. Of this species, 45.5% of the isolates were resistant to ampicillin, 31.8% to tetracycline, 27.3% to penicillin, and 18.2% to streptomycin, and for the remaining antibiotics, values ranged between 13.6% and 4.5%.

In contrast to the mentioned *S. aureus* isolates, those of *S. epidermidis* showed similar behaviors, and all were resistant to both ampicillin and penicillin, and susceptible to vancomycin, gentamicin, nitrofurantoin, and clindamycin. The percentages of isolates resistant to tetracycline (33.3%) and ciprofloxacin (16.7%) were like those obtained for *S. aureus* isolates. For the remaining species, the results were unequal, but the low number of isolates assayed does not allow any conclusion to be drawn.

PCR analysis for antibiotic-resistant genes (Table 2) showed that only four of the 13 genes assayed were present: *tet*M gene, encoding for tetracycline resistance, was present in 54.3% of the isolates; *erm*B and *erm*C genes, both encoding erythromycin resistance, were in 42.9% and 17.1% of the isolates, respectively; *grl*A gene, encoding ciprofloxacin resistance, was in 20% of the isolates. It was noted that *mec*A gene, encoding methicillin resistance, was not detected in any of the isolates assayed.

Results for the different species showed that while *grl*A gene was not detected in any *S. epidermidis* isolate, *erm*B gene was present in 100% of them. *tet*M gene was detected in 54.5% of the *S. aureus* isolates and in 66.7% of the *S. epidermidis* and *S. arlettae* isolates.

The prevalence of genes varied by isolate. An amount of 17.1% (isolates Sa03, Sa14, Sa19, Sa20, Sa21, Ss01) did not harbor any of the genes; 40% harbored one resistance gene, and 42.9% harbored two or three genes. The isolates harboring the highest number of genes were Sa01, Sa11, and Se05.

Concordance between phenotypic and genetic results was obtained for vancomycin and gentamicin. In addition, 63.6% of the isolates resistant to tetracycline in the phenotypic assay harbored *tet*M gene; 40% of those resistant to ciprofloxacin harbored *grl*A gene, and all the isolates resistant to erythromycin harbored *erm*B or *erm*C genes. However, discrepancies in the results from both analyses were also observed. The most remarkable was what occurred for β-lactams, because none of the 16 isolates resistant to ampicillin and penicillin harbored any of the genes encoding for resistance to β-lactams assayed (*bla*TEM, *oxa*, and *mec*A).

### 3.3. Biogenic Amine Production

**Figure 1:**
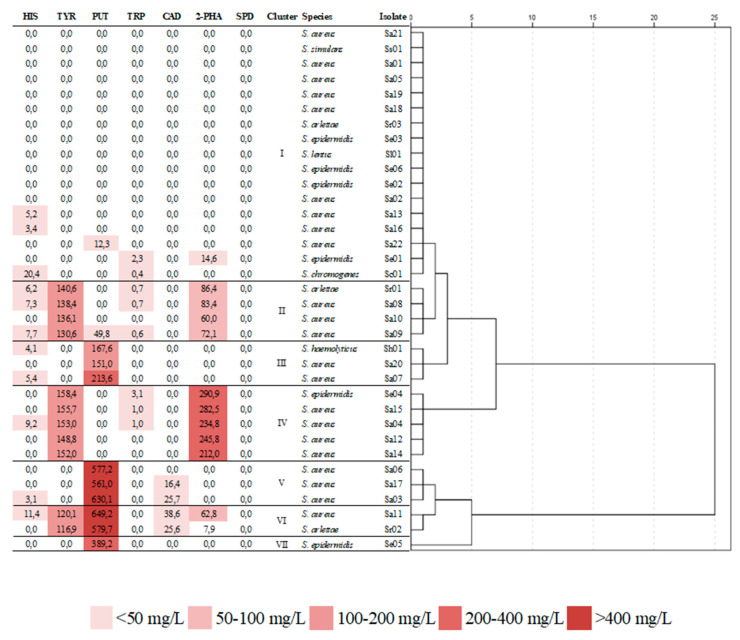
Abridged dendrogram of *Staphylococcus* strains according to their biogenic amine profile. HIS = histamine, TYR = tyramine, PUT = putrescine, TRP = tryptamine, CAD = cadaverine, 2-PHA = 2-phenylethylamine, SPD = spermidine.

The isolates of *Staphylococcus* evaluated in this study produced different (*p* < 0.05) amounts of BAs (Figure 1). Seventeen of the 35 isolates (47%) produced very low amounts of BAs, with a maximum total BA production below 32 mg/L. These isolates belonged to the species *S. aureus* (nine isolates), *S. epidermidis* (four isolates), *S. chromogenes* (one isolate), *S. lentus* (one isolate), *S. arlettae* (one isolate), and *S. simulans* (one isolate). On the other hand, the remaining isolates (53%) produced at least one of the amines analyzed in significantly (*p* < 0.05) greater amounts, with a total BA production higher than 150 mg/L, in some cases reaching concentrations exceeding 600 mg/L for a single amine.

Specifically, the isolates that showed the highest aminobiogenic capacity were, in decreasing order, Sa11, Sr02, Sa03, Sa06, Sa17, Se04, Sa15, Sa12, Sa04, and Se05, belonging to the species *S. aureus*, *S. arlettae*, and *S. epidermidis*. The most produced BA by far was putrescine, in concentrations ranging between 12.3 and 649.2 mg/L, followed by 2-phenylethylamine (between 7.9 and 290.9 mg/L), and tyramine (between 116.9 and 158.4 mg/L). None of the isolates produced spermidine, and few produced low concentrations of histamine and barely detectable amounts of tryptamine.

Figure 1 shows the dendrogram obtained from the Hierarchical Cluster Analysis (HCA) of the BAs, which shows the grouping of isolates into seven clusters. One cluster is constituted by both non-amine-producing and very low-amine-producing isolates, and included *S. aureus*, *S. simulans*, *S. epidermidis*, *S. arlettae*, *S. lentus*, and *S. chromogenes* isolates. Clusters II and IV comprise isolates that produced moderate (cluster II) and high (cluster IV) amounts of both tyramine and 2- phenylethylamine, and included *S. aureus*, *S. epidermidis*, and *S. arlettae* isolates. Cluster III comprises moderate putrescine-producing isolates, and clusters V, VI, and VII include high putrescine-producing isolates.

PCR reactions for the detection of tyrosine decarboxylase (*tdc*), histidine decarboxylase (*hdc*), ornithine decarboxylase (*odc*), and lysine decarboxylase (*ldc*) genes revealed that none of the isolates harbored any of these genes.

### 3.4. Relationships between Farming Practices and Antibiotic Resistance and Biogenic Amine Production by Staphylococci

The main farm management practices collected from the farmers’ responses to the questionnaire are presented in Table 4.

The nonlinear canonical correlation (OVERALS) between farming practices and antibiotic resistance and BA production are shown in Table 5. Eigenvalues, canonical correlation coefficients, and fit values were set to show the similarities among groups of variables.

**Figure 2:**
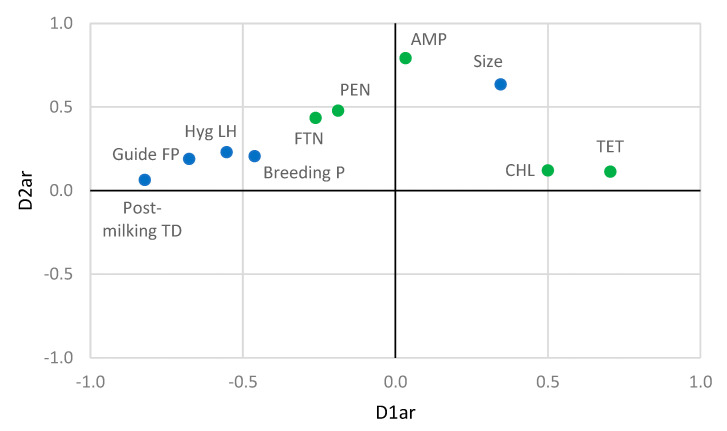
Nonlinear canonical correlation analysis similarity map determined by the first (D1ar) and second dimension (D2ar) for farming practices (●) and antibiotic resistances (●). Leyend: Size = flock size, Breeding P = Breeding program, Guide FP = guide to good farming practices, Hyg LH = hygiene of the livestock housing, Postmilking TD = Postdipping teat disinfection, AMP = ampicillin, PEN = penicillin, TET = tetracycline, FTN = nitrofurantoin, CHL = chloramphenicol.

The relationship between farming practices and antibiotic resistance obtained the best fit with the two-dimensional solution. The first dimension had a high explanatory power with an eigenvalue greater than 0.8, while the second dimension had a slightly lower explanatory power with an eigenvalue greater than 0.7. The two dimensions produced a fit of 1.648 and explained 82.4% of the variation in the model. The first dimension had a canonical correlation of 0.789 and related resistance to TET and CHL, with Hyg LH, Guide FP, Breeding P, and Postdipping TD (Figure 2). The risk of finding staphylococci resistant to TET and CHL in milk decreased when the farm was enrolled in a Breeding P, applied both a Guide FP and a Postdipping TD, and had better Hyg LH.

The second dimension had a canonical correlation of 0.577 and related resistance to AMP, PEN, and FTN with Size. The risk of finding staphylococci resistant to AMP, PEN, and FTN in milk decreased in smaller flocks.

**Figure 3:**
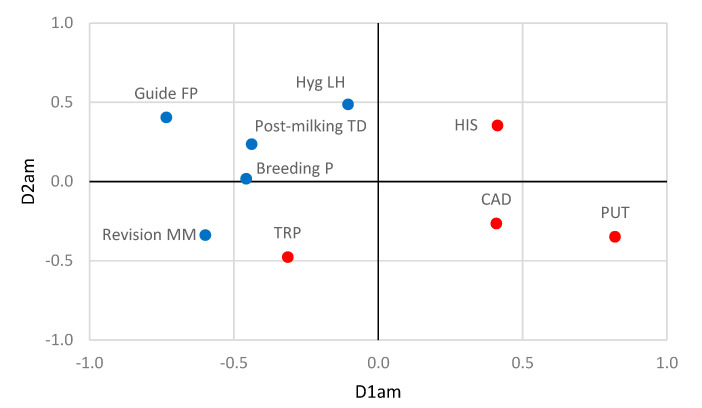
Nonlinear canonical correlation analysis similarity map determined by the first (D1am) and second dimension (D2am) for farming practices (●) and biogenic amines (●). Leyend: Breeding P = Breeding program, Guide FP = guide to good farming practices, Hyg LH = hygiene of the livestock housing, Postmilking TD = Postdipping teat disinfection, Revision MM = periodic check-up of the milking machine, HIS = histamine, PUT = putrescine, TRP = tryptamine, CAD = cadaverine.

The relationship between farming practices and the BA production obtained the best fit with the two-dimensional solution. The two dimensions had eigenvalues greater than 0.7, indicating a quite high explanatory power, and produced a fit of 1.544, explaining 77.2% of the variation in the model. The first dimension had a canonical correlation of 0.617 and related the presence of HIS, PUT, and CAD to the application of a Guide FP, Revision MM, Breeding P, and Postdipping TD (Figure 3). The risk of finding HIS, PUT, and CAD in milk increased when the Guide FP was not applied, the milking machine was not periodically checked, Postdipping TD was not applied, and the farm was not enrolled in a Breeding P. The second dimension had a canonical correlation of 0.472 and related the presence of TRP with the Hyg LH and the application of a Guide FP. The risk of finding TRP in milk increased when a Guide FP was not applied, and the Hyg LH was inadequate.

## 4. Discussion

Staphylococci counts were highly variable for samples from different farms, in concordance with the reported by other authors [31,32]. This fact could be related to differences in farming practices and could have important consequences from the perspective of both animal health (risk of infection, increase in animal replacement, and decrease in milk production, both leading to significant economic losses) and public health (antibiotic resistance, BA production, etc.).

The staphylococcal contamination profile in bulk tank ewe’s milk from this study revealed the presence of species that cause both clinical mastitis (*S. aureus*) and subclinical mastitis (coagulase-negative staphylococci, especially *S. epidermidis*), and it agrees with the results found by other authors in sheep milk [2,33,34].

Results from the antibiotic resistance phenotypic assay revealed a high percentage of isolates resistant to many of the antibiotics assayed, which could have repercussions for public health. Sarmah et al. [35] reported that the use of antibiotics by the livestock industry, as a preventive rather than a curative measure, could have contributed to the appearance of resistant strains, and to relieve this situation, EU has regulated use of antimicrobials in livestock (Regulation EU no 2019/6 [11]). In addition, the improvement of the protocols and the establishment of authorized products for the treatment of intramammary infections in sheep would be desirable, especially those produced by staphylococci, as was performed in cows [36]. Mavrogianni et al. [37] recommend carrying out an antibiogram prior to the administration of specific antimicrobials against the causative agent at drying off.

Incidence of resistance to antibiotics varied with the species and the isolates, but results obtained for the different antibiotics were sometimes coincident with those reported by other authors. As in this study, Rahmdel et al. [38] reported that 100% of staphylococci from goat and sheep milk were susceptible to gentamicin, vancomycin, and chloramphenicol, while Ruiz et al. [19] reported, for staphylococci from goat milk, that 98% and 100% of strains were susceptible to vancomycin and nitrofurantoin, respectively, and only 10% were intermediate to chloramphenicol. Concordance with the results by Ed-Dra et al. [39] was obtained for gentamicin, vancomycin, ampicillin, and penicillin, though these authors reported higher percentages of *S. aureus* strains resistant to tetracycline (76.2%), chloramphenicol (19.1%), and streptomycin (84.1%).

As mentioned for the phenotypic assay, the prevalence of antibiotic-resistant genes varied with the isolates. Discrepancies in the results by Rahmdel et al. [38] were observed, since these authors displayed that *tet*M gene was present in only 18% of the Staphylococcus strains from goat and sheep milk, while *erm*B and *mec*A genes were present in the 85.7% of them. None of the isolates assayed in our study harbored the *mec*A gene encoding methicillin resistance. Ruiz et al. [19] reported low prevalence of antibiotic resistance genes for staphylococci from goat milk, with only two genes encoding for erythromycin resistance being present—the *erm*B and *mef*A genes. The presence of staphylococci harboring antibiotic resistance genes is a matter of concern because of the possibility of transmission to other bacteria through the food chain.

When results from phenotypic and genetic assays were compared, both concordances, as occurred for vancomycin, gentamicin, tetracycline, ciprofloxacin, and erythromycin, and discrepancies, as for β-lactams, between both types of analysis, were obtained. Other authors [19,20,38] also reported discrepancies that were explained by the presence of silent genes, by genetic mutations in the coding or promoter regions of the genes, or because the detection by PCR of a single gene in an antibiotic resistance operon may overlook the absence of the other genes involved in phenotypic expression [20].

In this context, knowledge of the associations of certain farming practices with the isolation of staphylococci would help to understand how these microorganisms behave in relation to their environments. Results from this study have revealed that the risk of finding staphylococci resistant to TET and CHL in milk decreases when the farm is enrolled in a Breeding P, has applied a Guide FP and a Postdipping TD, and the Hyg LH is better. In general, these factors are related to good farming practices, and they are included in the guides to good practice recommended to improve the hygiene of primary production [40].

Furthermore, farms enrolled in a Breeding P use standardized action protocols, which cover the recommendations set out in the guides to good practices in farms. These protocols are established at different levels, such as the reproductive level, the general management level, and the sanitary treatment level, which, in addition to contributing to the genetic improvement of the farm, affect the composition of ewes’ milk, allowing differentiation of enrolled farms from those that did not enroll [41].

On the other hand, the application of Postdipping TD is a proven tool for decreasing microbial and somatic cell counts in milk [13], and therefore, it could also contribute to reducing the presence of antibiotic-resistant staphylococci.

Another factor influencing the risk of finding AMP, PEN, and FTN resistant staphylococci in milk is the size of the flock; this risk is lower in smaller flocks. This could be a consequence of the higher counts obtained in samples from larger farms, as also reported previously [42], and it is especially interesting because β-lactams antibiotics, such as ampicillin and penicillin, are commonly used in dairy sheep farming for the treatment of mastitis. The lack of effectiveness of these antibiotics could have a significant impact on the control of mastitis, especially on farms where large flocks exist, because the size of the farm and the large number of animals make their management more difficult. This would lead to problems in animal health (increased incidence of disease and chronic diseases, etc.), with the corresponding economic consequences (death/slaughter of animals, increased cost of other treatments and veterinary assistance, etc.), and in public health (potential increase in antibiotic-resistant bacteria in the food chain) [1].

The different behavior showed by the *Staphylococcus* isolates in terms of the BA production (Figure 1) is in concordance with that described by other authors [43], who reported that amino acid decarboxylase activity can vary considerably at the isolate level. This result allows confirmation that BA production is an isolate-specific property, as reported previously [44]. The isolates producing the highest concentrations of BAs were those in clusters IV, V, and VI in Figure 1, which belonged to *S. aureus*, *S. arlettae*, and *S. epidermidis* species, and the most produced BAs were putrescine and 2- phenylethylamine. Even et al. [20] also reported the production of 2-phenylethylamine and putrescine as major amines by some *Staphylococcus* species, such as *S. carnosus* and *S. epidermidis*. It should be noted that isolates that produced both tyramine and 2-phenylethylamine did not produce putrescine (clusters II and IV), and vice versa (clusters III, V and VII), with the only exception of the two isolates in cluster VI. The simultaneous production of tyramine and 2-phenylethylamine has been previously reported, and it has also been observed that 2-phenylethylamine generally occurs when a high amount of tyramine is present [45].

Varied results on the production of BAs by staphylococcal isolates have been reported. Müller et al. [46] investigated the production of BAs by RP-HPLC of 22 antibiotic-sensitive and nontoxigenic *S. carnosus* isolates and reported that none of them produced cadaverine, putrescine, or histamine under the experimental conditions used, although 12 isolates produced 2-phenylethylamine in concentrations ranging between 2.6 and 15.0 mg/L, which were much lower than those found in the present study. On the contrary, Bermúdez et al. [43] reported concentrations of putrescine of 1415 and 977 mg/L for two isolates of *S. equorum* and *S. epidermidis*, respectively, as well as cadaverine in a concentration of 36 mg/L for both isolates. It should be highlighted that some of the *Staphylococcus* isolates in our study produced remarkably high amounts of BAs, which could affect the quality of milk if the environmental conditions favor their growth and could have a negative impact on the quality of artisanal cheeses.

On the other hand, the fact that none of the isolates harbored any of the decarboxylase genes—*tdc*, *hdc*, *odc*, and *ldc*—assayed is in concordance with results from different authors [19,20] for staphylococci from fermented foods and goat milk. However, these results do not correspond with those from the phenotypic assay. Jeong et al. [44] affirmed that BA production is not linked to the existence of a specific amino acid decarboxylase gene, and Even et al. [20] reported that the lack of correlation between BA production and presence of genetic determinants could be due to the use of nonstaphylococcal species primers in the PCR reactions.

The high variability found for the production of BAs among staphylococcal isolates could be influenced by certain farming practices, although the food safety authorities [6] have not implemented measures to limit such incidence as they have for the use of antibiotics. Our results suggest that the risk of production of some BAs, particularly HIS, PUT, and CAD, in milk decreases when the farm is enrolled in a Breeding P and correctly applies the Guide FP, including a periodic check of the milking machine and an adequate Postdipping TD. In the same way, the risk of finding TRP in milk decreased when a Guide FP was applied and the Hyg LH was adequate. Therefore, the production of raw milk with the lowest possible staphylococcal counts should be considered a so-called “performance objective” in a holistic strategy, aimed at the control of production of BAs via the implementation of a food safety objective (FSO) approach [47].

As with the presence of antibiotic-resistant isolates, BA production is influenced by hygiene practices on farms and the sanitary conditions during milk production and storage, because both affect the staphylococcal contamination. Therefore, the adoption on dairy sheep farms of practices such as periodic checks of the milking machine or Postdipping TD would help to reduce the risk of the presence of BAs in milk. Furthermore, the influence of farms’ application of a Guide FP joined with a Breeding P demonstrates that the planning of farming practices is necessary in any area of the production system [42].

## 5. Conclusions

From the results of this study, it should be highlighted that among the isolates comprising the indigenous staphylococcal population in bulk tank ewe´s milk, there are multidrug-resistant strains harboring some resistance genes which have the potential to be dangerous. The aminobiogenic capacity of some of these isolates is also a trait with a significant impact on consumer health, and therefore, it would be advisable to both limit the staphylococci population in raw milk and avoid conditions that favor the production of these BAs.

In addition, it was demonstrated that certain practices used on farms, especially those related to hygienic-sanitary conditions, affect the risk of finding antibiotic-resistant and BA-producing *Staphylococcus* isolates in milk. Therefore, farmers should make stronger efforts not only to prevent animals from suffering an intramammary infection, but also to carry out good milk production practices in order to improve its microbiological quality, to avoid compromising consumers’ health, and to stop the spread of antibiotic-resistant bacteria.

## Figures and Tables

**Table 1:** Primers used for detection of genes assayed in this study.

Target Gene	Primer Sequence (5′-3′)	Product Size (bp *)	Annealing Temperature (°C)
Antibiotic resistance			
*bla*TEM	AGGAAGAGTATTCAACA	635	55
	CTCGTCGTTTGGTATGG		
*Oxa*	GTCTTTCAGAG-TACGGCATTA	822	55
	GATTTTCTTAGCGGCAACTTA		
*mec*A	TGGCTCAGGTACTGCTATCC	533	50
	CACCTTGTCCGTAACCTGAA		
*van*A	ATGAATAGAATAAAAGTTGC	1032	62
	TCACCCCTTTAACGCTAATA		
*tet*M	GTGTGACGAACTTTACCGAA	501	55
	GCTTTGTATCTCCAAGAACAC		
*gyr*A	AATGAACAAGGTATGACACC	618	55
	TACGCGCTTCAGTATAACGC		
*grl*A	ACTTGAAGATGTTTTAGGTGAT	618	55
	TTAGGAAATCTTGATGGCAA		
*aac6′/aph2″*	CCAAGAGCAATAAGGGCATA	220	45
	CACTATCATAACCACTACCG		
*erm*A	GTTCAAGAACAATCAATACAGAG	421	52
	GGATCAGGAAAAGGACATTTTAC		
*erm*B	CCGTTTACGAAATTGGAACAGGTAAAGGGC	359	55
	GAATCGAGACTTGAGTGTGC		
*erm*C	GCTAATATTGTTTAAATCGTCAATTCC	572	52
	GGATCAGGAAAAGGACATTTTAC		
*mef*A	ACTATCATTAATCACTAGTGC	346	55
	TTCTTCTGGTACTAAAAGTGG		
*msr*A	GGCACAATAAGAGTGTTTAAAGG	940	50
	AAGTTATATCATGAATAGATTGTCCTGTT		
	CGTGTTTTCAACATTTAATGCAA		
Biogenic amines			
*tdc*	ACATAGTCAACCATRTTGAA	1133	52
	CAAATGGAAGAAGAAGTAGG		
*hdc*	GATGGTATTGTTTCKTATGA	435	52
	CCAAACACCAGCATCTTC		
*odc*	NCAYAARCAACAAGYNGG	900	52
	GRTANGGNTNNGCACCTTC		
*ldc*	CAYRTNCCNGGNCAYAA	1185	53
	GGDATNCCNGGNGGRTA		

* base pair.

**Table 2:** Results of phenotypic (Phen) and genotypic (Gen) antibiotic resistance assays.

		β-Lactams					Glycopeptides		Tetracyclines		Quinolones			Aminoglucosides			Macrolides							Others	
Species	Isolate	Phen		Gen			Phen	Gen	Phen	Gen	Phen	Gen		Phen	Phen	Gen	Phen	Phen	Gen					Phen	Phen
		AMP	PEN	*bla*TEM	*oxa*	*mec*A	VAN	*van*A	TET	*tet*M	CIP	*gyr*A	*grl*A	SMN	GMN	*aac6′/aph2″*	CMN	ERY	*erm*A	*erm*B	*erm*C	*mef*A	*msr*A	FTN	CHL
*S. aureus*	Sa01	S	S	−	−	−	S	−	R	+	S	−	+	I	S	−	S	S	−	+	−	−	−	S	S
	Sa02	S	S	−	−	−	S	−	S	+	S	−	−	S	S	−	S	I	−	−	−	−	−	S	S
	Sa03	S	S	−	−	−	S	−	S	−	S	−	−	R	S	−	I	I	−	−	−	−	−	S	S
	Sa04	S	S	−	−	−	S	−	S	−	S	−	−	S	S	−	S	I	−	+	−	−	−	S	S
	Sa05	S	S	−	−	−	S	−	S	+	S	−	−	S	S	−	S	S	−	−	+	−	−	S	S
	Sa06	R	R	−	−	−	S	−	R	+	S	−	−	R	S	−	R	I	−	+	−	−	−	R	S
	Sa07	S	S	−	−	−	S	−	R	+	S	−	−	I	S	−	S	I	−	+	−	−	−	S	S
	Sa08	R	R	−	−	−	S	−	S	−	S	−	−	S	S	−	I	S	−	+	−	−	−	S	S
	Sa09	S	S	−	−	−	S	−	R	+	S	−	−	S	S	−	R	R	−	−	+	−	−	S	S
	Sa10	R	R	−	−	−	S	−	S	+	I	−	+	R	S	−	S	I	−	+	−	−	−	S	S
	Sa11	S	S	−	−	−	S	−	S	+	R	−	+	I	S	−	I	I	−	−	+	−	−	S	S
	Sa12	R	R	−	−	−	S	−	R	+	R	−	−	R	S	−	I	I	−	+	−	−	−	S	R
	Sa13	R	R	−	−	−	S	−	I	−	R	−	+	S	S	−	I	I	−	−	−	−	−	I	I
	Sa14	S	S	−	−	−	S	−	S	−	S	−	−	I	S	−	I	I	−	−	−	−	−	S	S
	Sa15	S	S	−	−	−	S	−	S	+	I	−	−	I	S	−	I	I	−	−	−	−	−	S	S
	Sa16	R	S	−	−	−	S	−	S	−	I	−	+	I	S	−	I	I	−	−	−	−	−	S	S
	Sa17	S	S	−	−	−	S	−	R	−	S	−	+	I	S	−	I	I	−	−	−	−	−	S	I
	Sa18	R	R	−	−	−	S	−	S	+	S	−	−	S	S	−	I	S	−	−	+	−	−	S	S
	Sa19	R	S	−	−	−	S	−	S	−	S	−	−	S	S	−	S	I	−	−	−	−	−	S	S
	Sa20	S	S	−	−	−	S	−	S	−	S	−	−	S	S	−	S	S	−	−	−	−	−	S	S
	Sa21	R	S	−	−	−	S	−	S	−	S	−	−	S	S	−	S	S	−	−	−	−	−	S	S
	Sa22	R	S	−	−	−	S	−	R	+	S	−	−	S	S	−	S	S	−	−	−	−	−	S	S
*S. epidermidis*	Se01	R	R	−	−	−	S	−	R	−	S	−	−	S	S	−	S	S	−	+	−	−	−	S	S
	Se02	R	R	−	−	−	S	−	S	−	I	−	−	S	S	−	S	S	−	+	−	−	−	S	S
	Se03	R	R	−	−	−	S	−	R	+	S	−	−	S	S	−	S	S	−	+	−	−	−	S	S
	Se04	R	R	−	−	−	S	−	S	+	R	−	−	S	S	−	S	S	−	+	−	−	−	S	R
	Se05	R	R	−	−	−	S	−	S	+	S	−	−	R	S	−	S	I	−	+	−	−	−	S	S
	Se06	R	R	−	−	−	S	−	S	+	S	−	−	S	S	−	S	R	−	+	−	−	−	S	S
*S. arlettae*	Sr01	S	R	−	−	−	S	−	S	−	I	−	+	R	S	−	S	I	−	+	−	−	−	R	S
	Sr02	R	R	−	−	−	S	−	I	+	S	−	−	S	S	−	I	S	−	−	−	−	−	S	S
	Sr03	R	R	−	−	−	S	−	S	+	S	−	−	S	S	−	I	S	−	−	−	−	−	S	S
*S. lentus*	Sl01	S	S	−	−	−	S	−	R	−	S	−	−	S	S	−	I	I	−	−	+	−	−	S	S
*S. haemolyticus*	Sh01	S	S	−	−	−	S	−	S	+	R	−	−	S	S	−	S	I	−	−	+	−	−	S	S
*S. simulans*	Ss01	R	R	−	−	−	S	−	S	−	S	−	−	S	S	−	S	S	−	−	−	−	−	S	S
*S. chromogenes*	Sc01	R	R	−	−	−	S	−	R	−	S	−	−	S	S	−	I	R	−	+	−	−	−	S	S

AMP = ampicillin; PEN = penicillin; VAN = vancomycin; TET = tetracycline; CIP = ciprofloxacin; SMN = streptomycin; GMN = gentamicin; CMN = clindamycin; ERY = Erythromycin; FTN = nitrofurantoin; CHL = chloramphenicol. S = susceptible; I = intermediate; R = resistant.

**Table 3:** Multiple antimicrobial resistance (MAR) index.

Species	Isolate ^a^	Antibiotic Resistance ^b^	MAR Index
*S. aureus*	Sa01	TET	0.09
	Sa03	SMN	0.09
	Sa06	AMP, PEN, TET, FTN, CMN, SMN	0.54
	Sa07	TET	0.09
	Sa08	AMP, PEN	0.18
	*S*a09	TET, ERY, CMN	0.27
	Sa10	AMP, PEN, SMN	0.09
	Sa11	CIP	0.09
	Sa12	AMP, PEN, TET, CIP, SMN, CHL.	0.54
	Sa13	AMP, PEN, CIP	0.27
	Sa16	AMP	0.09
	Sa17	TET	0.09
	Sa18	AMP, PEN	0.18
	Sa19	AMP	0.09
	Sa21	AMP	0.09
	Sa22	AMP, TET	0.18
* S. epidermidis *	Se01	AMP, PEN, TET	0.27
	Se02	AMP, PEN	0.18
	Se03	AMP, PEN, TET	0.27
	Se04	AMP, PEN, CIP, CHL	0.36
	Se05	AMP, PEN, SMN	0.27
	Se06	AMP, PEN, ERY	0.27
* S. arlettae *	Sr01	PEN, FTN, SMN	0.27
	Sr02	AMP, PEN	0.18
	Sr03	AMP, PEN	0.18
* S. lentus *	Sl01	TET	0.09
* S. haemolyticus *	Sh01	CIP	0.09
* S. simulans *	Ss01	AMP, PEN	0.18
* S. chromogenes *	Sc01	AMP, PEN, TET, ERY	0.36

^a^ Isolates that were susceptible or intermediate have not been included. ^b^ AMP = ampicillin, PEN = penicillin, TET = tetracycline, CIP = ciprofloxacin, ERY = Erythromycin, FTN = nitrofurantoin, CMN = clindamycin, SMN = streptomycin, CHL = chloramphenicol.

**Table 4:** Distribution of frequencies obtained in the applied questionnaire.

Variable	Levels	Frequency (%)
Size	<1300	22 (62.9)
	≥1300	13 (37.1)
Breeding P	Not enrolled	18 (51.4)
	Enrolled	17 (48.6)
Guide FP	No	12 (34.3)
	Yes	23 (65.7)
Hyg LH	Not	18 (51.4)
	Adequate	17 (48.6)
Hyg MP	Not	20 (57.1)
	Adequate	15 (42.9)
Milkline	Mid	21 (60.0)
	Low	14 (40.0)
M Stalls	<48	13 (37.1)
	≥48	22 (62.9)
Ewes/M Stalls	<30	22 (62.9)
	≥30	13 (37.1)
Revision MM	None	18 (51.4)
	Periodic	17 (48.6)
Acid	Less frequently	28 (80.0)
	Daily	7 (20.0)
M Vaccination	No	25 (71.4)
	Yes	10 (28.6)
Postmilking TD	Not	4 (11.4)
	Always	31 (88.6)
Dry-off T	No	21 (60.0)
	Yes	14 (40.0)

Leyend: Size = flock size, Breeding P = Breeding program, Guide FP = guide to good farming practices, Hyg LH = hygiene of the livestock housing, Hyg MP = hygiene of the milking parlour, Milkline = milkline of the milking machine, M Stalls = milking stalls, Ewes M Stalls = ewes per milking stall, Revision MM = periodic check-up of the milking machine, Acid = frequency of use of acid for cleaning the milking machine, M Vaccination = mastitis vaccination, Postmilking TD = Postdipping teat disinfection, Dry-off T = Dry-off treatment.

**Table 5:** The component loadings of variables in the analysis of nonlinear canonical correlation analysis (OVERALS).

Set	Variable	D1ar	D2ar	D1am	D2am
Farming practices	Size	0.345	0.635	-	-
	Breeding P	−0.462	0.206	−0.458	0.019
	Guide FP	−0.676	0.190	−0.734	0.405
	Hyg LH	−0.553	0.229	−0.105	0.488
	Hyg MP	-	-	-	-
	Milkline	-	-	-	-
	M Stalls	-	-	-	-
	Ewes/M Stalls	-	-	-	-
	Revision MM	-	-	−0.599	−0.338
	Acid	-	-	-	-
	M Vaccination	-	-	-	-
	Postmilking TD	−0.822	0.064	−0.439	0.236
	Dry-off T	-	-	-	-
Antibiotic resistances	AMP	0.033	0.792		
	PEN	−0.188	0.477		
	VAN	-	-		
	TET	0.704	0.113		
	CIP	-	-		
	SMN	-	-		
	GMN	-	-		
	CMN	-	-		
	ERY	-	-		
	FTN	−0.262	0.435		
	CHL	0.499	0.121		
Amines	HIS			0.413	0.354
	TYR			-	-
	PUT			0.820	−0.348
	TRP			−0.313	−0.476
	CAD			0.409	−0.265
	2-PHA				
	SPD				
Eigenvalue		0.860	0.720	0.808	0.736
Canonical *r*		0.789	0.577	0.617	0.472

Leyend: Size = flock size, Breeding P = Breeding program, Guide FP = guide to good farming practices, Hyg LH = hygiene of the livestock housing, Hyg MP = hygiene of the milking parlour, Milkline = milkline of the milking machine, M Stalls = milking stalls, Ewes M Stalls = ewes per milking stall, Revision MM = periodic check-up of the milking machine, Acid = frequency of use of acid for cleaning the milking machine, M Vaccination = mastitis vaccination, Postmilking TD = Postdipping teat disinfection, Dry-off T = Dry-off treatment, AMP = ampicillin, PEN = penicillin, VAN = vancomycin TET = tetracycline, CIP = ciprofloxacin, SMN = streptomycin, GMN = gentamicin, CMN = clindamycin, ERY = Erythromycin, FTN = nitrofurantoin, CHL = chloramphenicol, HIS = histamine, TYR = tyramine, PUT = putrescine, TRP = tryptamine, CAD = cadaverine, 2-PHA = 2-phenylethylamine, SPD = spermidine.
